# *Post Hoc* Analysis of the Phase II DESTINY-PanTumor02 Study: Local and Central HER2 IHC Concordance and Trastuzumab Deruxtecan Efficacy by HER2 IHC Status in HER2-Expressing Solid Tumors

**DOI:** 10.1158/1078-0432.CCR-25-4702

**Published:** 2026-04-09

**Authors:** Vicky Makker, Jung-Yun Lee, Do-Youn Oh, Ana Oaknin, Soham Puvvada, Lindsey Jung, Robert McEwen, Flavia Michelini, Funda Meric-Bernstam

**Affiliations:** 1 Gynecologic Medical Oncology Service, Memorial Sloan Kettering Cancer Center, New York, New York.; 2 Department of Obstetrics and Gynecology, Yonsei Cancer Center and Severance Hospital, Yonsei University College of Medicine, Seoul, Republic of Korea.; 3 Department of Internal Medicine, Seoul National University Hospital, Cancer Research Institute, Seoul National University College of Medicine, Integrated Major in Innovative Medical Science, Seoul National University Graduate School, Seoul, Republic of Korea.; 4 Medical Oncology Service, Vall d’Hebron Institute of Oncology (VIHO), Hospital Universitari Vall d’Hebron, Vall d’Hebron Barcelona Hospital Campus, Barcelona, Spain.; 5 Clinical Development, Late Oncology, Oncology R&D, AstraZeneca, Gaithersburg, Maryland.; 6 Oncology Biometrics, Oncology R&D, AstraZeneca, Gaithersburg, Maryland.; 7 Oncology Data Science, Research and Early Development, Oncology R&D, AstraZeneca, Cambridge, United Kingdom.; 8 Translational Medicine, Oncology R&D, AstraZeneca, Barcelona, Spain.; 9 Department of Investigational Cancer Therapeutics, The University of Texas MD Anderson Cancer Center, Houston, Texas.

## Abstract

**Purpose:**

Accurate HER2 status assessment is crucial to identify patients with HER2-expressing tumors. In this *post hoc* analysis of DESTINY-PanTumor02 (NCT04482309), we report concordance between local and central HER2 immunohistochemistry (IHC) results.

**Patients and Methods:**

This open-label, phase II study evaluated trastuzumab deruxtecan (T-DXd; 5.4 mg/kg once every 3 weeks) for HER2-expressing (IHC 3+/2+ by local or central testing) locally advanced, unresectable, or metastatic solid tumors after ≥1 systemic treatment or without alternative treatments.

**Results:**

In total, 267 patients received T-DXd; 75.7% were enrolled based on local test results. Concordance between local and central HER2 test results was 58.6% for IHC 3+, 54.5% for IHC 2+, and 73.4% for IHC 3+ and IHC 2+ tumors combined.

**Conclusions:**

The moderate concordance observed between local and central IHC testing in DESTINY-PanTumor02 highlights the need for a validated diagnostic test and standardized algorithm for HER2 status assessment in solid tumors to ensure appropriate patient identification for HER2-directed therapies.

**Translational Relevance**

At the time of the study, American Society of Clinical Oncology/College of American Pathology guidelines for HER2 scoring were only available for breast and gastric cancers. This *post hoc* analysis indicated that although the current HER2 IHC testing effectively identifies patients who benefit from treatment, validated diagnostic tests and standardized algorithms for the assessment of HER2 status in other solid tumors could enhance patient identification for HER2-directed therapies and optimize treatment outcomes.

## Introduction

HER2 overexpression has been detected in a range of solid tumors, beyond breast and gastric tumors (1), and may be associated with poor prognosis and an aggressive tumor phenotype (2, 3).

Trastuzumab deruxtecan (T-DXd) is an antibody–drug conjugate composed of a humanized immunoglobulin G1 monoclonal antibody specifically targeting HER2, a tetrapeptide-based cleavable linker, and a potent topoisomerase I inhibitor payload (4, 5).

T-DXd is approved in numerous countries for various indications, including HER2-positive [immunohistochemistry (IHC) 3+, IHC 2+/*in situ* hybridization-positive (ISH+)], HER2-low (IHC 1+, IHC 2+/ISH-negative), and HER2-ultralow (IHC 0 with membrane staining) breast cancer; HER2-positive (IHC 3+, IHC 2+/ISH+) gastric or gastroesophageal junction adenocarcinoma; and *HER2*-mutant non–small cell lung cancer (NSCLC; refs. 6, 7). Furthermore, T-DXd is also approved in multiple countries, including the United States and the United Kingdom, for the treatment of adult patients with unresectable or metastatic HER2-positive (IHC 3+) solid tumors that have progressed after prior treatment and have no satisfactory alternative treatment options (6, 8, 9).

To determine HER2 status and enable appropriate patient identification for potential HER2-directed therapy, American Society of Clinical Oncology/College of American Pathology (ASCO/CAP) guidelines are available for breast and gastric cancers (10, 11). These guidelines provide recommendations on sample examination, staining methodology, distinguishing IHC scores, and performing ISH testing (10, 11). However, there are currently no standardized guidelines for other tumor types. Moreover, in clinical settings, HER2 IHC and ISH testing may be conducted at different sites (local or central) with varying testing practices (12), which may contribute to variable concordance rates between local and central testing laboratories (13).

In part 1 of the two-part open-label phase II DESTINY-PanTumor02 trial, T-DXd demonstrated clinically meaningful benefit in pretreated patients with HER2-expressing solid tumors, with the greatest benefit observed in those with HER2 IHC 3+ expression by central test result (14). The safety profile was consistent with the known profile for T-DXd, including the incidence of interstitial lung disease/pneumonitis (14). In DESTINY-PanTumor02, HER2 expression for study enrollment was based on local or central IHC test results (14), and a recent *post hoc* analysis demonstrated clinically meaningful activity with T-DXd irrespective of whether HER2 status was determined by local or central testing at enrollment (15).

To gain insights into HER2 testing practices and their potential impact on clinical outcomes, we report concordance rates between local and central HER2 IHC test results and a *post hoc* efficacy analysis according to local or central HER2 IHC test results from DESTINY-PanTumor02.

## Patients and Methods

### Study design

DESTINY-PanTumor02 (NCT04482309) is a two-part, open-label, phase II study evaluating T-DXd (5.4 mg/kg once every 3 weeks) for HER2-expressing locally advanced or metastatic disease after ≥1 systemic treatment or without alternative treatments. Part 1 study design details and outcome measures have been previously published (14).

### Patients

Eligible patients were female or male, aged 18 years or older, with histologically confirmed locally advanced, unresectable, or metastatic biliary tract, bladder, cervical, endometrial, ovarian, pancreatic, or other solid tumors (excluding breast, NSCLC, gastric, and colorectal cancers) that had progressed following prior treatment or had no satisfactory alternative treatment options.

HER2 expression for enrollment was based on a local IHC test result where available; otherwise, enrollment was determined using a central IHC test result. Scoring was based on current ASCO/CAP guidelines for scoring HER2 in gastric cancer (11), irrespective of local or central IHC testing. Antibody systems used for local testing were required to be CAP/Clinical Laboratory Improvement Amendments compliant and included the Roche HER2 4B5 (RRID: AB_2335975; Roche Diagnostics), HercepTest (RRID: AB_2935822; Dako, Agilent), Bond Oracle HER2 IHC System (Leica Biosystems), or other lab-developed tests that met this requirement. Patients who were enrolled based on a local test result also had HER2 expression determined by retrospective central testing using the HercepTest, with scoring according to gastric-specific criteria (11). Mandatory formalin-fixed, paraffin-embedded tumor samples obtained at the time of diagnosis of metastatic or locally advanced, unresectable disease (most recent preenrollment tumor sample) were provided; local and central testing was performed on samples from the same tissue block, where possible. Samples must have been no older than 3 years (for patients enrolled after February 2021; older samples were permitted prior to this protocol amendment). Pathologists performing central HER2 IHC testing were blinded to the local HER2 IHC test results.

### Procedures

T-DXd was administered intravenously once every 3 weeks at 5.4 mg/kg of body weight until documented disease progression [Response Evaluation Criteria in Solid Tumors (RECIST) 1.1], withdrawal of consent, or if any other discontinuation criteria were met. Owing to the single-arm, open-label design of part 1 of the DESTINY-PanTumor02 study, no randomization or blinding was performed.

### Endpoints

The primary endpoint was the confirmed objective response rate (ORR) by investigator assessment (per RECIST 1.1). Secondary endpoints included duration of response (DOR), progression-free survival (PFS), overall survival (OS), and safety. An independent central review according to RECIST 1.1 was conducted alongside the investigator-assessed results for secondary outcomes. Exploratory endpoints included subgroup analyses by HER2 status (IHC 3+ vs. IHC 2+ vs. IHC 1+ vs. IHC 0, as reported here).

In this analysis, concordance was calculated as the percentage of samples with the same IHC score by both local and central test results and further analyzed by tumor type, tissue block age (number of days between sample collection and study enrollment), local test type (HercepTest vs. Roche HER2 4B5 assay), sample type [incision biopsy (biopsy) vs. excision biopsy (resection)], and tumor type (primary vs. metastatic); the PATHWAY anti-HER2/neu (4B5) Rabbit Monoclonal Primary Antibody (RRID: AB_2335975; Roche Diagnostics), available in some regions as the VENTANA HER2 (4B5) Rabbit Monoclonal Primary Antibody RxDx (RRID: AB_2921204), will be hereafter referred to as the Roche HER2 4B5 assay. Images of HER2 IHC staining by local testing were not available for review; concordance was assessed according to the HER2 IHC test result provided by the local testing site.

### Study oversight

All patients provided written informed consent before participating in any study procedures. The study received approval from the independent Institutional Review Boards at each participating site and was conducted in accordance with the ethical principles of the Declaration of Helsinki and the Good Clinical Practice guidelines defined by the International Conference on Harmonisation.

## Results

As previously reported, a total of 268 patients with HER2-expressing solid tumors were enrolled from >120 sites between October 7, 2020, and July 7, 2022 (14). Of these patients, 267 (99.6%; *n/N* = 267/268) received at least one dose of T-DXd and were included in the full analysis set (14). Demographics and baseline clinical characteristics are summarized in Table 1, and the representativeness of the study population is described in Supplementary Table S1.

**Table 1.** Baseline characteristics.

	All (*N* = 267)	IHC 3+ by local test result (*n* = 93)	IHC 2+ by local test result (*n* = 107)	IHC 3+ by central test result (*n* = 75)	IHC 2+ by central test result (*n* = 125)
Median age, years (range)	62 (23–85)	66 (23–85)	60 (30–80)	64 (31–85)	62 (23–81)
Sex	​	​	​	​	​
Female	178 (66.7)	55 (59.1)	74 (69.2)	47 (62.7)	83 (66.4)
ECOG performance status^a^	​	​	​	​	​
0	126 (47.2)	51 (54.8)	49 (45.8)	42 (56)	54 (43.2)
1	140 (52.4)	42 (45.2)	57 (53.3)	33 (44)	71 (56.8)
Prior therapy lines	​	​	​	​	​
Median (range)	2 (0–12)	2 (0–9)	2 (1–12)	2 (0–9)	2 (1–12)
0	3 (1.1)	2 (2.2)	0	1 (1.3)	2 (1.6)
1	71 (26.6)	27 (29)	25 (23.4)	22 (29.3)	33 (26.4)
≥2	193 (72.3)	64 (68.8)	82 (76.6)	52 (69.3)	90 (72)
Tumor type	​	​	​	​	​
Biliary tract	41 (15.4)	18 (19.4)	16 (15)	16 (21.3)	14 (11.2)
Bladder	41 (15.4)	24 (25.8)	9 (8.4)	16 (21.3)	20 (16)
Cervical	40 (15)	8 (8.6)	13 (12.1)	8 (10.7)	20 (16)
Endometrial	40 (15)	14 (15.1)	17 (15.9)	13 (17.3)	17 (13.6)
Ovarian	40 (15)	13 (14)	24 (22.4)	11 (14.7)	19 (15.2)
Pancreatic	25 (9.4)	4 (4.3)	11 (10.3)	2 (2.7)	19 (15.2)
Other^b^	40 (15)	12 (12.9)	17 (15.9)	9 (12)	16 (12.8)
Prior HER2 therapy^c^	38 (14.2)	23 (25.3)	12 (11.2)	18 (24.3)	12 (9.7)

All values are *n* (%) unless stated otherwise. In the cervical cohort, five patients with IHC 1+ status were included after a protocol-specified interim analysis; for each cohort (except the other tumors cohort), up to 10 patients with IHC 1+ tumors by prospective central testing could have been included if ≥3 objective responses were observed in the first 15 patients with confirmed HER2 IHC 3+ or 2+ by central test result; for the other tumors cohort, only patients with HER2 IHC 3+ or 2+ tumors were eligible for enrollment.

Abbreviation: ECOG, Eastern Cooperative Oncology Group.

a One patient with HER2 IHC 2+ by the test result used for study enrollment was enrolled with an ECOG performance status of 2.

b Included: salivary gland cancer (*n* = 19), malignant neoplasm of unknown primary site (*n* = 5), extramammary Paget disease (*n* = 3), cutaneous melanoma (*n* = 2), oropharyngeal neoplasm (*n* = 2), and adenoid cystic carcinoma, head and neck cancer, lip and/or oral cavity cancer, esophageal adenocarcinoma, esophageal squamous cell carcinoma, testicular cancer, and vulvar carcinoma (all *n* = 1).

c Included trastuzumab, pertuzumab, zanidatamab, trastuzumab emtansine, trastuzumab duocarmazine, and tucatinib.

In total, 202 of 267 (75.7%) patients were enrolled based on a local HER2 IHC test result; of these, 93 (34.8%) and 107 (40.1%) patients had IHC 3+ and IHC 2+ tumors, respectively. Two (0.7%) patients with IHC 1+ tumors based on a local test result were included following a protocol-specified interim analysis. Of the >120 study sites, 48 used local test results for enrollment, with varying HER2 testing procedures used (Roche HER2 4B5 assay, *n* = 27; HercepTest, *n* = 9; Bond Oracle HER2 IHC System, *n* = 4; lab-developed test, *n* = 2; unknown, *n* = 6).

All patients who were enrolled based on a local HER2 test result also had their HER2 status evaluated by retrospective central testing, and an additional 65 of 267 (24.3%) patients were enrolled based on a central HER2 test result only. According to central test results (*N* = 267), 75 (28.1%) patients had IHC 3+ tumors, 125 (46.8%) patients had IHC 2+ tumors, 25 (9.4%) patients had IHC 1+ tumors, and 30 (11.2%) patients had IHC 0 tumors. HER2 status by central test was unknown for 12 (4.5%; ref. 14) patients, including patients whose samples were unevaluable (for various technical reasons) and may include patients for whom a sample was not provided for central testing.

### HER2 IHC local and central test result concordance

**Figure 1.**
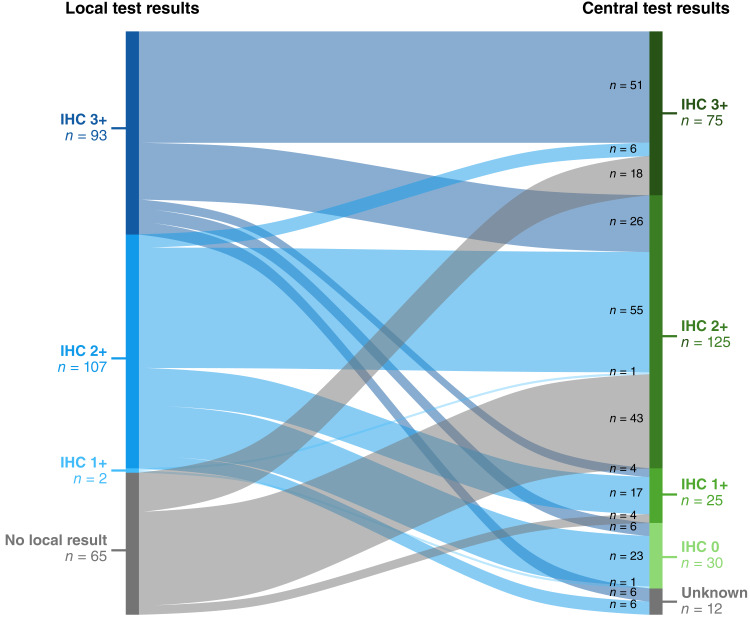
Concordance between local and central HER2 IHC test results. The protocol did not specify the use of specific antibodies for local testing. Central HER2 IHC testing utilized the HercepTest (Dako, Agilent), with samples scored according to gastric cancer–specific criteria. Unknown IHC status by central test result includes patients whose samples were unevaluable (for various technical reasons) and may include patients for whom a sample was not provided for central testing. Overall concordance between local and central HER2 results: IHC 3+, 58.6%; IHC 2+, 54.5%; and IHC 3+ and IHC 2+ combined, 73.4%.

Overall, the positive percentage agreement (PPA) between local and central HER2 test results was 58.6% [95% confidence interval (CI), 47.6–69.1; *n/N* = 51/87] for IHC 3+ tumors, 54.5% (95% CI, 44.2–64.4; *n/N* = 55/101) for IHC 2+ tumors, and 73.4% (95% CI, 66.5–79.6; *n/N* = 138/188) for IHC 3+ and IHC 2+ combined (Fig. 1; Table 2). Concordance rates for local and central HER2 IHC 3+ and 2+ varied across tumor cohorts, with the highest agreement between local and central HER2 test results reported for IHC 2+ bladder tumors (77.8%; 95% CI, 40–97.2; *n/N* = 7/9) and the lowest reported for IHC 3+ pancreatic tumors (25%; 95% CI, 0.6–80.6; *n/N* = 1/4; Table 2).

**Table 2.** Concordance^a^ rates between local and central HER2 test results and tumor type/cohort.

	Central HER2 result					
	IHC 3+	IHC 2+	IHC 1+	IHC 0	IHC unknown	Total
**Local HER2 result**	​	​	​	​	​	​
Overall	​	​	​	​	​	​
IHC 1+	0	1	0	1	0	2
IHC 2+	6	55 (54.5%; 44.2–64.4)	17	23	6	107
IHC 3+	51 (58.6%; 47.6–69.1)	26	4	6	6	93
Biliary tract	​	​	​	​	​	​
IHC 2+	0	7 (46.7%; 21.3–73.4)	2	6	1	16
IHC 3+	12 (66.7%; 41–86.7)	4	1	1	0	18
Bladder	​	​	​	​	​	​
IHC 2+	1	7 (77.8%; 40–97.2)	1	0	0	9
IHC 3+	12 (52.2%; 30.6–73.2)	8	1	2	1	24
Cervical	​	​	​	​	​	​
IHC 1+	0	1	0	1	0	2
IHC 2+	0	7 (53.8%; 25.1–80.8)	3	3	0	13
IHC 3+	6 (75%; 34.9–96.8)	1	1	0	0	8
Endometrial	​	​	​	​	​	​
IHC 2+	2	7 (43.8%; 19.8–70.1)	3	4	1	17
IHC 3+	9 (64.3%; 35.1–87.2)	3	1	1	0	14
Other tumors^b^	​	​	​	​	​	​
IHC 2+	1	7 (53.8%; 25.1–80.8)	2	3	4	17
IHC 3+	4 (57.1%; 18.4–90.1)	2	0	1	5	12
Ovarian	​	​	​	​	​	​
IHC 2+	2	12 (50%; 29.1–70.9)	5	5	0	24
IHC 3+	7 (53.8%; 25.1–80.8)	6	0	0	0	13
Pancreatic	​	​	​	​	​	​
IHC 2+	0	8 (72.7%; 39–94)	1	2	0	11
IHC 3+	1 (25%; 0.6–80.6)	2	0	1	0	4

Sixty-five patients were enrolled based on a central HER2 test result and had no local test result: biliary tract, *n* = 7; bladder, *n* = 8; cervical, *n* = 17; endometrial, *n* = 9; other tumors, *n* = 11; ovarian, *n* = 3; and pancreatic, *n* = 10.

a Concordance was defined as the percentage of samples with the same IHC score by both local and central test results; PPA was calculated excluding samples with an unknown IHC score by central test result. Results are reported as *n* (%; 95% CI).

b Included: salivary gland cancer (*n* = 19), malignant neoplasm of unknown primary site (*n* = 5), extramammary Paget disease (*n* = 3), cutaneous melanoma (*n* = 2), oropharyngeal neoplasm (*n* = 2), adenoid cystic carcinoma, head and neck cancer, lip and/or oral cavity cancer, esophageal adenocarcinoma, esophageal squamous cell carcinoma, testicular cancer, and vulvar carcinoma (all *n* = 1).

Concordance rates between central HER2 IHC test results by HercepTest and local Roche HER2 4B5 assay or local HercepTest results are detailed in Table 3. Of the patients enrolled based on a local HER2 test result, the Roche HER2 4B5 assay was used for 162 patients, HercepTest was used for 24 patients, and the local HER2 test was unknown for 16 patients. Concordance for IHC 2+ and IHC 3+ tumors was similar when HercepTest was used both locally and centrally (70%; 95% CI, 34.8–93.3; *n/N* = 7/10 and 72.7%; 95% CI, 39–94; *n/N* = 8/11 PPA, respectively), whereas agreement was numerically lower when the Roche HER2 4B5 assay was used locally (IHC 2+: 50.6%; 95% CI, 39.4–61.8; *n/N* = 42/83 and IHC 3+: 55.7%; 95% CI, 43.3–67.6; *n/N* = 39/70).

**Table 3.** Concordance^a^ rates between central HER2 HercepTest results and local HER2 HercepTest or Roche HER2 4B5 assay test results.

	Central HER2 HercepTest result					
	IHC 3+	IHC 2+	IHC 1+	IHC 0	IHC unknown^b^	Total
**Local HER2 HercepTest result**	​	​	​	​	​	​
IHC 1+	0	0	0	1	0	1
IHC 2+	0	7 (70%; 34.8–93.3)	2	1	1	11
IHC 3+	8 (72.7%; 39–94)	2	1	0	1	12
** Total**	**8**	**9**	**3**	**2**	**2**	**24**
**Local Roche HER2 4B5 assay result**	​	​	​	​	​	​
IHC 1+	0	2	0	0	0	2
IHC 2+	4	42 (50.6%; 39.4–61.8)	15	22	4	87
IHC 3+	39 (55.7%; 43.3–67.6)	22	3	6	3	73
** Total**	**43**	**64**	**18**	**28**	**7**	**162**

a Concordance was defined as the percentage of samples with the same IHC score by both local and central test results; PPA was calculated excluding samples with an unknown IHC status by central test result. Results are reported as *n* (%; 95% CI).

b Unknown IHC status by central test result includes patients whose samples were unevaluable (for various technical reasons) and may include patients for whom a sample was not provided for central testing.

In the analysis of central test results and local test results using biopsy or resection samples (Table 4), concordance was similar for IHC 3+ tumors (59.1%; 95% CI, 43.2–73.7; *n/N* = 26/44 for biopsies; 58.1%; 95% CI, 42.1–73; *n/N* = 25/43 for resection samples), whereas a numerically higher agreement was observed for IHC 2+ biopsy samples (61.5%; 95% CI, 47–74.7; *n/N* = 32/52) compared with IHC 2+ resection samples (46.9%; 95% CI, 32.5–61.7; *n/N* = 23/49).

**Table 4.** Concordance^a^ rates between central HER2 IHC test results and local test results according to biopsy or resection.

	Central HER2 test result					
	IHC 3+	IHC 2+	IHC 1+	IHC 0	IHC unknown^b^	Total^c^
**Local HER2 test result—biopsy**	​	​	​	​	​	​
IHC 1+	0	1	0	1	0	2
IHC 2+	3	32 (61.5%; 47–74.7)	8	9	4	56
IHC 3+	26 (59.1%; 43.2–73.7)	14	3	1	1	45
**Enrolled based on a central HER2 test result**	10	24	2	0	0	36
**Total**	**39**	**71**	**13**	**11**	**5**	**139**
**Local HER2 test result—resection**	​	​	​	​	​	​
IHC 1+	0	0	0	0	0	0
IHC 2+	3	23 (46.9%; 32.5–61.7)	9	14	2	51
IHC 3+	25 (58.1%; 42.1–73)	12	1	5	4	47
**Enrolled based on a central HER2 test result**	8	19	2	0	0	29
**Total**	**36**	**54**	**12**	**19**	**6**	**127**

a Concordance was defined as the percentage of samples with the same IHC score by both local and central test results; PPA was calculated excluding samples with an unknown IHC status by central test result. Results are reported as *n* (%; 95% CI).

b Unknown central IHC status by central test result includes patients whose samples were unevaluable (for various technical reasons) and may include patients for whom a sample was not provided for central testing.

c One patient did not provide any tumor sample.

When analyzing agreement between central HER2 test results and local HER2 test results by use of primary or metastatic tumor samples (Supplementary Table S2), the highest concordance was observed for IHC 3+ metastatic tumor samples (61.9%; 95% CI, 45.6–76.4; *n/N* = 26/42); a similar PPA was also observed for IHC 2+ metastatic tumor samples (61.1%; 95% CI, 46.9–74.1; *n/N* = 33/54). Concordance between local and central HER2 test results was numerically lower when a primary tumor sample was used (IHC 2+: 46.8%; 95% CI, 32.1–61.9; *n/N* = 22/47 and IHC 3+: 55.6%; 95% CI, 40–70.4; *n/N* = 25/45). A higher proportion of primary tumor samples were resections (65.4%; *n/N* = 83/136) compared with biopsies (38.1%; *n/N* = 53/136), whereas in metastatic tumor samples, biopsies comprised 61.9% (*n/N* = 86/130) and resections comprised 34.6% (*n/N* = 44/130).

Tissue blocks were obtained for 163 patients, with block identification information available for 43 patients who had both local and central HER2 test results. Among these patients with block identification information, the same tissue block was used for both local and central HER2 testing in 81% (*n/N* = 35/43) of cases.

The median sample age (time between biopsy collection and study enrollment) was similar for cases with concordant and discordant HER2 IHC scores, both overall and for individual IHC scores (Supplementary Fig. S1).

### Efficacy

At data cutoff (June 2023), investigator-assessed confirmed ORRs (95% CI) in patients with HER2 IHC 3+ and IHC 2+ tumors by local test result were 51.6% (41–62.1) and 24.3% (16.5–33.5), respectively. Investigator-assessed confirmed ORRs (95% CI) in patients with HER2 IHC 3+, IHC 2+, IHC 1+, and IHC 0 tumors by central test result were 61.3% (49.4–72.4), 27.2% (19.6–35.9), 24% (9.4–45.1), and 30% (14.7–49.4), respectively. Median DOR, PFS, and OS by local and central test results are reported in Table 5.

**Table 5.** Efficacy by local and central HER2 IHC test results.

	IHC 3+ by local test result	IHC 2+ by local test result	IHC 1+ by local test result	IHC 3+ by central test result^a^	IHC 2+ by central test result^a^	IHC 1+ by central test result^a^	IHC 0 by central test result
*N*	93	107	2	75	125	25	30
ORR,^b^ % (*n*) 95% CI	51.6 (48) 41–62.1	24.3 (26) 16.5–33.5	0	61.3 (46) 49.4–72.4	27.2 (34) 19.6–35.9	24 (6) 9.4–45.1	30 (9) 14.7–49.4
Median DOR,^b^^,^^c^ months 95% CI	22.1 10.6–23.6	10.3 4.5–14.1	—	22.1 9.6–NE	9.8 4.3–12.6	14.2 8.3–NE	9.9 2.6–NE
Median PFS,^b^ months 95% CI	9.9 7–12.5	5.1 3.5–6	4.2 2.6–NE	11.9 8.2–13	5.4 4.2–6	6.9 1.4–9.7	4.2 3–7.1
Median OS, months 95% CI	17.7 12.6–23.4	10.7 8–13	6.8 6.1–NE	21.1 15.3–29.6	12.2 10.7–13.5	9.2 6.4–NE	7.6 5.1–13

Abbreviation: NE, not evaluable.

a Includes patients enrolled by central HER2 IHC test result and those evaluated by retrospective central testing.

b By investigator assessment.

c DOR includes only patients with an objective response.

## Discussion

This analysis examined the concordance between local and central HER2 test results in solid tumors across seven cohorts of the DESTINY-PanTumor02 study. Moderate concordance was observed between local and central HER2 test results overall (IHC 3+: 58.6%; IHC 2+: 54.5%; IHC 3+ and IHC 2+ combined: 73.4%). Across individual tumor types, there was variation in concordance rates, ranging from 25% for IHC 3+ pancreatic tumors to 77.8% for IHC 2+ bladder tumors; however, small sample sizes in some cohorts limit the interpretation of these results. Despite the variation in agreement observed, T-DXd antitumor activity was similar in patients who had their HER2 IHC status evaluated by a local or central IHC test. Taken together, these data reinforce the need for accurate HER2 testing, both locally and centrally, in solid tumors to ensure appropriate identification of patients who may benefit from HER2-directed therapy.

Accurate assessment of HER2 status is crucial for the effective treatment of many HER2-expressing tumors (16). The use of trastuzumab in breast cancer highlighted the importance of validated HER2 testing and scoring to accurately identify patients likely to benefit from treatment (17), and this observation remains important and has broader implications following the recent approval of T-DXd for the treatment of HER2-positive (IHC 3+) solid tumors (6, 8, 9). HER2 testing and scoring protocols differ across cancer types; for example, testing in gastric cancer contrasts with breast cancer testing owing to inherent differences in tumor biology, such as HER2 heterogeneity (focal staining) and incomplete membrane staining, which are more frequently observed in gastric tumors (18). Beyond breast and gastric cancers, there may be additional differences observed across other tumor types, which could affect testing. Indeed, the CAP recently published updated guidance for HER2 testing in endometrial cancers, which was not available at the time of patient enrollment. This guidance describes similar HER2 staining patterns in endometrial cancers as those reported in gastric cancers, with tumor cells exhibiting basolateral/lateral membrane staining and frequent heterogeneity (19). The expanding use of HER2-directed therapies across multiple tumor types, combined with emerging evidence of subtle differences in HER2 staining patterns, highlights the need for standardized scoring guidelines for HER2 testing.

Variability in concordance rates has been seen across various studies in breast and gastric cancers (13, 20–22). In breast cancer, concordance rates are generally high (78%–92%) and have shown notable improvements over time with the implementation of standardized guidelines, validated assays, and increased experience among pathologists (13, 22), whereas more variation is reported in gastric cancers (64%–88% agreement), which could be attributed to high tumoral heterogeneity (20, 21). In this study, the observed concordance was generally lower compared with previous reports, potentially influenced by tumor heterogeneity, interpathologist variability, regional testing differences, and variation in the HER2 antibodies used (12, 22–24). Laboratory standardization and quality control are, therefore, crucial aspects of HER2 IHC testing that ensure accurate and reproducible results across different facilities, including the use of validated protocols that align with the current ASCO/CAP guidelines, and the use of FDA-approved tests and automated novel testing methods to minimize technical variability (25).

Our results are consistent with findings from a recent analysis describing concordance between three HER2 IHC scoring algorithms across multiple tumor types, in which low agreement was observed between three independent pathologists, particularly when scoring IHC 2+ and IHC 1+ tumors (26). These results highlighted the reality of interpathologist variability and indicated that greater awareness of best scoring practices is needed to ensure reliable identification of patients likely to benefit from treatment with T-DXd (26). It should also be noted that the ASCO/CAP scoring algorithms for gastric and breast cancer were comparable in their identification of IHC 3+ tumors (26), increasing confidence that patients with IHC 3+ tumors were correctly identified.

Concordance between local and central HER2 test results did not seem to be specific to tumor type or sample age. However, concordance may have been affected by the local HER2 test used and the sample type used for local HER2 testing. For example, PPA was numerically higher when a HercepTest was used in a local laboratory compared with the use of a Roche HER2 4B5 assay; however, these findings should be interpreted with caution given the small number of patients who had a local HercepTest result. Interestingly, the HercepTest has previously been shown to detect HER2 expression with higher sensitivity than the Roche HER2 4B5 assay in breast cancers with *HER2* amplification or HER2-low status, which warrants further investigation into whether higher assay sensitivity will indeed improve patient selection for HER2-directed therapies (27). Notably, concordance for the HER2 IHC 2+ subgroup was approximately 15% higher when a biopsy sample was used for local HER2 testing compared with local testing using resection samples, and was approximately 14% higher for local HER2 testing using metastatic tissue compared with local testing using a primary tumor sample. Prior studies investigating HER2 testing using biopsy samples have reported evidence to suggest that the smaller sample size may allow for more complete fixation and better antigen preservation compared with resection samples (28). Additionally, the smaller sample size of a biopsy allows for analysis of the entire tissue within a single field of view, which may enable pathologists to better evaluate tissue HER2 expression compared with larger resection samples that require scanning across multiple fields. Although the smaller sample size of a biopsy may offer some advantages, issues with the representation of tumor HER2 heterogeneity have been previously reported, which may influence concordance rates (28). It should also be noted that the scoring guidelines for HER2 IHC in gastric cancers differ between resection and biopsy samples. In resection specimens, a score of IHC 3+, IHC 2+, or IHC 1+ requires strong complete membranous staining, weak-to-moderate complete membranous staining, or faint membranous staining, respectively, in ≥10% of tumor cells. Conversely, in biopsy specimens, IHC 3+/2+/1+ expression is defined by the presence of these staining patterns in a cluster of ≥5 tumor cells, irrespective of the overall percentage of positive cells (11), which could have also influenced concordance rates. Interestingly, a higher proportion of metastatic tumor samples were biopsies versus resections, potentially affecting the concordance observed; however, there was not 100% agreement between tumor type (primary vs. metastatic) and sample type (biopsy vs. resection). Together, these findings may have implications for future testing protocols, although further research is necessary to confirm these findings.

In line with the efficacy findings reported in this analysis, an additional *post hoc* analysis of DESTINY-PanTumor02 previously demonstrated the antitumor activity of T-DXd regardless of whether HER2 expression was evaluated by a central or local HER2 test at enrollment, with particular benefit in patients with IHC 3+ tumors (15). Of note, responses were also observed in patients with IHC 3+ or 2+ tumors by local test result whose tumors were reclassified as HER2 IHC 1+ or 0 by central testing. However, considering the moderate concordance rates observed herein, a validated clinical diagnostic test and a standardized algorithm for HER2 testing in solid tumors would facilitate the accurate identification of patients most likely to benefit therapeutically.

This tumor-agnostic study has inherent limitations, including the single-arm design, which does not enable the inclusion of comparators given the range of tumor cohorts investigated. The relatively small sample size, particularly when considering the broad range of tumor cohorts, may limit the interpretation of outcomes by subgroup. In addition, study enrollment was based on local or central IHC analysis of HER2 status, and 75.7% of patients were enrolled based on a local test result, the majority of which used the Roche HER2 4B5 assay (80.2%). Furthermore, there were 16 patients for whom the local HER2 testing procedure was not reported. Moreover, the use of the ASCO/CAP guidelines for scoring HER2 in gastric cancer for local and central HER2 IHC assessment was defined in the protocol; however, there may have been instances in which alternative guidelines were used, affecting concordance rates. Finally, there were challenges associated with obtaining detailed information on local tests (such as block ID and surgery date) that was more readily available for central tests, which may affect the interpretation of some analyses.

In this *post hoc* analysis of the DESTINY-PanTumor02 study, moderate concordance was observed between local and central HER2 IHC testing. Notably, the clinical benefit of T-DXd was demonstrated in patients with HER2-expressing solid tumors regardless of whether HER2 IHC status was determined by local or central testing. These findings suggest that while current HER2 IHC testing methods effectively identify patients who benefit from treatment, further development of validated diagnostic tests and standardized assessment algorithms may enhance patient identification and optimize treatment outcomes.

## Supplementary Material

Supplementary Data 1 Supplement

## Data Availability

Data underlying the findings described in this article may be obtained in accordance with AstraZeneca’s data sharing policy described at https://www.astrazenecaclinicaltrials.com/our-transparency-commitments. Data for studies directly listed on Vivli can be requested through Vivli at www.vivli.org. Data for studies not listed on Vivli can be requested through Vivli at https://vivli.org/members/enquiries-about-studies-not-listed-on-the-vivli-platform/. The AstraZeneca Vivli member page is also available, outlining further details: https://vivli.org/ourmember/astrazeneca/.
