# Supplementary material for: Post Hoc Analysis of the Phase II DESTINY-PanTumor02 Study: Local and Central HER2 IHC Concordance and Trastuzumab Deruxtecan Efficacy by HER2 IHC Status in HER2-Expressing Solid Tumors
Source: Clin Cancer Res. 2026 Apr 9;32(13):2628–36. doi: 10.1158/1078-0432.CCR-25-4702 (PMC13320193; doi:10.1158/1078-0432.CCR-25-4702)
Supplement: Supplementary Data 1 — Supplement [file ccr-25-4702_supplementary_data_1_suppsd_1.docx]

**SUPPLEMENTARY APPENDIX**

***
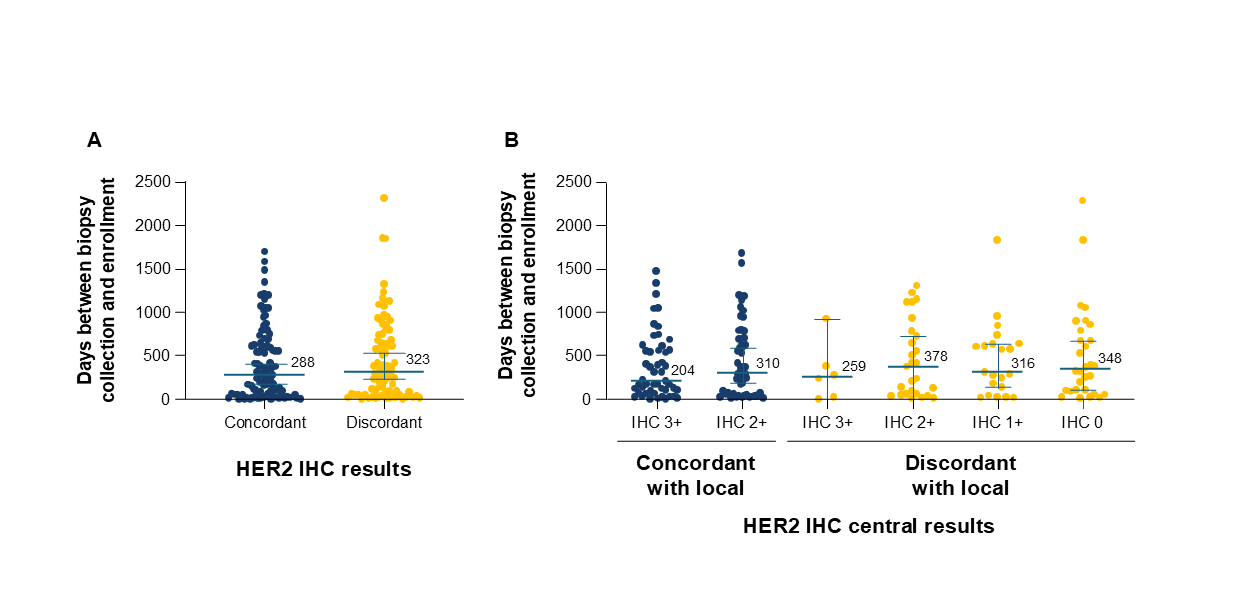
*Supplementary Figure S1. Tissue block age of samples with concordant and discordant HER2 IHC scores overall (A) and by IHC status (B)**

Black horizontal bars represent median age (days), and error bars represent 95% confidence intervals

Samples were classified as concordant if the IHC score was the same by local and central test result; samples were discordant if the IHC scores differed by local and central test results. Patients without a local test result were excluded from the analysis.

IHC, immunohistochemistry.

**Supplementary Table S1.** Representativeness of study participants

| **Population characteristic** | **Description** |
| --- | --- |
| Cancer type(s)/subtype(s)/stage(s)/condition | HER2-expressing (IHC 3+/2+) solid tumors: endometrial (*n* =40), cervical (*n* = 40), ovarian (*n* = 40), bladder (*n* = 41), biliary tract cancer (*n* = 41), pancreatic (*n* = 25), and other (*n* = 40)* |
| Considerations related to: | |
| Sex | Endometrial and cervical cancers occur only in individuals with a uterus. Ovarian cancer occurs only in individuals with ovaries.  Bladder cancer is more frequently observed among males compared with females; the most recent global incidence estimates reported an age-standardized rate of 9.3% in males versus 2.4% in females (1).  The incidence of biliary tract cancer differs by sex depending on the primary tumor location; intrahepatic bile duct cancers are more common in males (age-standardized rate of 12.7% versus 4.8%; combined rate reported for liver and intrahepatic bile duct cancers), whereas gallbladder cancers are more predominant in females (1.4% versus 0.9%) (1).  Pancreatic cancer affects both males and females, with a slight trend towards higher incidence among males (age-standardized rate of 5.5% versus 4.0%) (1). |
| Age | Approximate median age at diagnosis of endometrial cancer is 64 years, cervical cancer is 50 years, ovarian cancer is 63 years, bladder cancer is 73 years, biliary tract cancer is 70 years, and pancreatic cancer is 71 years (2–4). |
| Race/ethnicity | Incidence of endometrial cancer is higher in non-Hispanic White individuals compared with other racial or ethnic groups (5). Ovarian cancer is more frequently reported in non-Hispanic American Indian / Alaska Native (AI/AN) individuals and Hispanic individuals of any race (6). Similarly, cervical cancer is frequently observed in non-Hispanic AI/AN and Hispanic (any race) women (6).  Bladder cancer has the highest incidence in non-Hispanic White individuals compared with other racial or ethnic groups (6).  Incidence of biliary tract cancer is highest in non-Hispanic White individuals compared with any other racial or ethnic group (7).  The highest incidence rates of pancreatic cancer are reported among non-Hispanic Black individuals compared with other racial or ethnic groups (6). |
| Geography | Incidence of endometrial cancer is highest in North America and Europe compared with other regions; the lowest incidence rates are reported in Africa and Asia (8,9). Incidence of ovarian cancer is also highest across Europe (Central, Eastern, and Northern Europe); the lowest incidence rates are reported in Central and Southern Africa, and the Caribbean (9,10). Unlike endometrial and ovarian cancers, the highest incidence rates of cervical cancer are reported across Eastern, Western, Southern, and Middle Africa, and Melanesia, while the lowest incidence rates are reported in North Africa, North America, Southern Europe, Western Asia, and Australia and New Zealand (1).  Incidence of bladder cancer is highest in Southern, Northern and Western Europe; the lowest incidence rates are reported in Middle Africa for males and in South-Central Asia for females (1).  Incidence of biliary tract cancer is highest across Asia-Pacific and South America, and lowest across Europe and North America (11).  Incidence of pancreatic cancer is highest across Europe and North America, while the lowest incidence rates are reported in Eastern, Western, and Middle Africa, and South-Central Asia (1). |
| Other considerations | HER2 IHC 3+ expression is reported in 4–17% of endometrial cancers (12–16), 4–6% of ovarian cancers (12,17,18), 8–14% of cervical cancers (12,19,20), 4–31% of bladder cancers (12,21–27), and 1–7% of pancreatic cancers (12,28,29). HER2 IHC 2+ expression is reported in 11–39% of endometrial cancers (12–16), 0–28% of ovarian cancers (12,17,18), 14–18% of cervical cancers (12,19,20), 5–55% of bladder cancers (12,21–26,30), and 6–9% of pancreatic cancers (12,28,29). |
| Overall representativeness of the study | The DESTINY-PanTumor02 study was conducted in 268 patients across 15 countries. The median age of participants in each tumor cohort was generally consistent with the values reported in the literature. The majority of participants enrolled into this study were White; Black individuals were less well represented among pancreatic cancer cohort, although incidence has been reported to be higher in these individuals. |

*Evaluation of the representativeness of the ‘other’ tumors cohort is limited by the broad range of tumor types included and the small sample sizes of each tumor type; therefore, no comments have been made regarding this cohort.

HER2, human epidermal growth factor receptor 2; IHC, immunohistochemistry.

**Supplementary Table S2. Concordance* rates between central HER2 IHC test results and local test results according to primary or metastatic tumor sample**

|  | | **Central HER2 test result** | | | | | |
| --- | --- | --- | --- | --- | --- | --- | --- |
|  |  | **IHC 3+** | **IHC 2+** | **IHC 1+** | **IHC 0** | **IHC unknown**^†^ | **Total**^‡^ |
| **Local HER2 test result – primary tumors** | **IHC 1+** | 0 | 1 | 0 | 1 | 0 | 2 |
|  | **IHC 2+** | 3 | 22 (46.8%)  [32.1–61.9] | 8 | 14 | 1 | 48 |
|  | **IHC 3+** | 25 (55.6%)  [40.0–70.4] | 13 | 3 | 4 | 5 | 50 |
| **Enrolled based on a central HER2 test result** | | 7 | 27 | 2 | 0 | 0 | 36 |
| **Total** | | **35** | **63** | **13** | **19** | **6** | **136** |
| **Local HER2 test result – metastatic tumors** | **IHC 1+** | 0 | 0 | 0 | 0 | 0 | 0 |
|  | **IHC 2+** | 3 | 33 (61.1%)  [46.9–74.1] | 9 | 9 | 5 | 59 |
|  | **IHC 3+** | 26 (61.9%)  [45.6–76.4] | 13 | 1 | 2 | 0 | 42 |
| **Enrolled based on a central HER2 test result** | | 11 | 16 | 2 | 0 | 0 | 29 |
| **Total** | | **40** | **62** | **12** | **11** | **5** | **130** |

*Concordance defined as the percentage of samples with the same IHC score by both local and central test result; positive percentage agreement was calculated excluding samples with an unknown IHC status by central test result. Results are reported as n (%) [95% CI].

^†^Unknown IHC/ISH status by central test result includes patients whose samples were unevaluable (for various technical reasons) and may include patients for whom a sample was not provided for central testing.

^‡^Primary or metastatic tumor sample information was not available for one patient.

CI, confidence interval; IHC, immunohistochemistry; ISH, in situ hybridization.

**REFERENCES**

1. Bray F, Laversanne M, Sung H, Ferlay J, Siegel RL, Soerjomataram I, et al. Global cancer statistics 2022: GLOBOCAN estimates of incidence and mortality worldwide for 36 cancers in 185 countries. CA Cancer J Clin. 2024;74:229–63.

2. Tarney CM, Tian C, Wang G, Dubil EA, Bateman NW, Chan JK, et al. Impact of age at diagnosis on racial disparities in endometrial cancer patients. Gynecol Oncol. 2018;149:12–21.

3. SEER Cancer Stat Facts [Internet]. SEER. [cited 2026 Jan 28]. Available from: https://seer.cancer.gov/statfacts/index.html

4. Wen W, Mumma M, Zheng W. Temporal trends of stages and survival of biliary tract cancers in the United States and associations with demographic factors. Cancer Epidemiol Biomarkers Prev. 2023;32:1660–7.

5. Clarke MA, Wentzensen N, Köbel M, Erickson BK, Wijayabahu AT, Kahn AG, et al. Age-specific incidence and five-year relative survival of endometrial cancer histotypes by race and ethnicity among US women, 2000 to 2019. Gynecologic Oncology. Elsevier; 2025;200:137–44.

6. SEER*Explorer Application [Internet]. [cited 2024 May 23]. Available from: https://seer.cancer.gov/statistics-network/explorer/application.html?site=612&data_type=4&graph_type=2&compareBy=sex&chk_sex_3=3&chk_sex_2=2&relative_survival_interval=5&race=1&age_range=1&stage=106&advopt_precision=1&advopt_show_ci=on&hdn_view=0&advopt_show_apc=on&advopt_display=1

7. Kim D, Manikat R, Wijarnpreecha K, Cholankeril G, Ahmed A. Burden of mortality from hepatocellular carcinoma and biliary tract cancers by race and ethnicity and sex in US, 2018–2023. Clin Mol Hepatol. 2024;30:756–70.

8. Qiu S, Jiang S, Ye Q, Yang Y, Li X. Global trends and geographical disparities in the incidence of uterine cancer from 1990 to 2021. European Journal of Obstetrics & Gynecology and Reproductive Biology. 2025;311:114066.

9. World Health Organization. Cancer Today [Internet]. [cited 2026 Feb 11]. Available from: https://gco.iarc.who.int/today/

10. Huang J, Chan WC, Ngai CH, Lok V, Zhang L, Lucero-Prisno DE, et al. Worldwide Burden, Risk Factors, and Temporal Trends of Ovarian Cancer: A Global Study. Cancers (Basel). 2022;14:2230.

11. Baria K, De Toni EN, Yu B, Jiang Z, Kabadi SM, Malvezzi M. Worldwide Incidence and Mortality of Biliary Tract Cancer. Gastro Hep Adv. 2022;1:618–26.

12. Uzunparmak B, Haymaker C, Raso G, Masciari S, Wang L, Lin H, et al. HER2-low expression in patients with advanced or metastatic solid tumors. Ann Oncol. 2023;34:1035–46.

13. Semiz H, Pala E, Can B, Atag E, Gungor H, Sanci M. cERBB-2/Her-2 Neu overexpression and prognostic significance in uterine carcinosarcoma. Turk Patoloji Derg. 2023;39:55–63.

14. Halle MK, Tangen IL, Berg HF, Hoivik EA, Mauland KK, Kusonmano K, et al. HER2 expression patterns in paired primary and metastatic endometrial cancer lesions. Br J Cancer. 2018;118:378–87.

15. Krakstad C, Berg HF, Lindemann K, Halle MK. Frequency of ERBB2-Low Expression in Endometrial Cancer. JAMA Oncol. 2024;10:1587–8.

16. van Dijk D, Vermij L, León-Castillo A, Powell M, Jobsen J, Leary A, et al. Clinical and Molecular Characteristics of High-Risk, Recurrent, or Metastatic Endometrial Cancer That Is Human Epidermal Growth Factor Receptor 2-Low. J Clin Oncol. 2025;43:443–52.

17. Chung YW, Kim S, Hong JH, Lee JK, Lee NW, Lee YS, et al. Overexpression of HER2/HER3 and clinical feature of ovarian cancer. J Gynecol Oncol. 2019;30:e75.

18. Kim Y-N, Chung YS, Park E, Lee ST, Lee J-Y. Human epidermal growth factor receptor-2 expression and subsequent dynamic changes in patients with ovarian cancer. Sci Rep. Nature Publishing Group; 2024;14:7992.

19. Shi H, Shao Y, Lu W, Lu B. An analysis of HER2 amplification in cervical adenocarcinoma: correlation with clinical outcomes and the International Endocervical Adenocarcinoma Criteria and Classification. J Pathol Clin Res. 2021;7:86–95.

20. Panek G, Ligaj M. Prognostic significance of HER-2/neu expression in patients at early clinical stages of invasive cervical cancer. Gin Onkol. 2007;5:218–35.

21. Fleischmann A, Rotzer D, Seiler R, Studer UE, Thalmann GN. HER2 amplification is significantly more frequent in lymph node metastases from urothelial bladder cancer than in the primary tumours. Eur Urol. 2011;60:350–7.

22. Gårdmark T, Wester K, De La Torre M, Carlsson J, Malmström P-U. Analysis of HER2 expression in primary urinary bladder carcinoma and corresponding metastases. BJU Int. 2005;95:982–6.

23. Moustakas G, Kampantais S, Nikolaidou A, Vakalopoulos I, Tzioufa V, Dimitriadis G. HER-2 overexpression is a negative predictive factor for recurrence in patients with non-muscle-invasive bladder cancer on intravesical therapy. J Int Med Res. SAGE Publications Ltd; 2020;48:0300060519895847.

24. Moktefi A, Pouessel D, Liu J, Sirab N, Maille P, Soyeux P, et al. Reappraisal of HER2 status in the spectrum of advanced urothelial carcinoma: a need of guidelines for treatment eligibility. Mod Pathol. Elsevier; 2018;31:1270–81.

25. Moradi Tabriz H, Nazar E, Ahmadi SA, Azimi E, Majidi F. Survivin and Her2 Expressions in Different Grades of Urothelial Neoplasms of Urinary Bladder. Iranian Journal of Pathology. 2020;16:154–61.

26. Koshkin VS, Schafer JM, Scherrer E, Boyiddle C, Schwartz NRM, Yu H, et al. Testing and Interpretation of Human Epidermal Growth Factor Receptor 2 Protein Expression and ERBB2 Gene Amplification in Advanced Urothelial Carcinoma. JCO Precis Oncol. Wolters Kluwer; 2025;e2400879.

27. Nam W, Chae HK, Jung Y, Kang H, Park M, Choi A, et al. HER2 positivity predicts BCG unresponsiveness and adaptive immune cell exhaustion in EORTC risk-stratified cohort of bladder cancer. Front Immunol. 2023;14:1301510.

28. Chou A, Waddell N, Cowley MJ, Gill AJ, Chang DK, Patch A-M, et al. Clinical and molecular characterization of HER2 amplified-pancreatic cancer. Genome Med. 2013;5:78.

29. Han S-H, Ryu KH, Kwon A-Y. The prognostic impact of HER2 genetic and protein expression in pancreatic carcinoma-HER2 protein and gene in pancreatic cancer. Diagnostics (Basel). 2021;11:653.

30. Nam W, Chae HK, Jung Y, Kang H, Park M, Choi A, et al. HER2 positivity predicts BCG unresponsiveness and adaptive immune cell exhaustion in EORTC risk-stratified cohort of bladder cancer. Front Immunol [Internet]. Frontiers; 2023 [cited 2025 Nov 28];14. Available from: https://www.frontiersin.org/journals/immunology/articles/10.3389/fimmu.2023.1301510/full
